# Inclusion of unqualified cacao pod in mineral feed block: a novel strategy to improve rumen fermentation and mitigate methane emissions in beef cattle

**DOI:** 10.5713/ab.25.0436

**Published:** 2025-10-22

**Authors:** Wuttikorn Srakaew, Chanon Suntara, Tanyatip Jittaniramon, Ratchanee Bourapa, Apichaya Feepakpro, Supanida Thongpun, Chantira Wongnen, Tansiphorn Na Nan

**Affiliations:** 1 Department of Animal Science and Fisheries, Faculty of Science and Agricultural Technology, Rajamangala University of Technology Lanna, Nan, Thailand; 2 Department of Animal Science, Faculty of Agriculture, Khon Kaen University, Khon Kaen, Thailand; 3 School of Agricultural Resources, Chulalongkorn University, Nan, Thailand; 4 Food Technology and Innovation Research Center of Excellence, School of Agricultural Technology and Food Industry, Walailak University, Nakhon Si Thammarat, Thailand; 5 The Innovation of Sustainable Thai Cacao Development Research Unit, Chulalongkorn University, Bangkok, Thailand

**Keywords:** Carbon Dioxide, Greenhouse Gas, Methane, Tannin, Unqualified Cacao Pod

## Abstract

**Objective:**

This study aimed to evaluate the effects of incorporating unqualified cacao pod powder into beef cattle diets on nutrient digestibility, rumen fermentation, greenhouse gas emissions, and blood metabolites.

**Methods:**

A 4×4 Latin square design was used with four of Brahman×Thai native crossbred steers (207.1±45.1 kg body weight). Treatments included; T1: a control (no supplement), T2: supplemented with 50 g/d of cacao powder, T3: supplemented with mineral block containing cacao powder and T4: both supplemented with 50 g/d of cacao powder and mineral block containing cacao powder.

**Results:**

Results showed no significant effect on feed intake, but polyphenol and tannin intake increased (p<0.01). Apparent digestibility of dry matter, organic matter, protein, and both detergent fibers increased with cacao supplementation (p<0.01). Rumen pH, total volatile fatty acid, and acetate concentrations increased, while methane and carbon dioxide emissions were reduced (p<0.01). Blood urea nitrogen levels decreased (p<0.05), while concentrations of white blood cells, red blood cells, hemoglobin, and lymphocyte percentage remained unchanged. The neutrophil percentage tended to decrease, as same as, the neutrophil to lymphocyte ratio in the supplemented groups (p = 0.06 and p = 0.08, respectively).

**Conclusion:**

These findings suggest that unqualified cacao pods can enhance nutrient utilization and mitigate enteric methane emissions, offering a sustainable strategy for valorizing cacao byproducts in ruminant production.

## INTRODUCTION

Cacao (*Theobroma cacao* L.) is an economically important crop worldwide. However, its cultivation is highly vulnerable to climate change [1,2], particularly in Thailand. Many Thai cacao farmers still lack sufficient knowledge and expertise in the proper cultivation and management of cacao trees, resulting in suboptimal yields. Cacao is highly sensitive to drought; its growth and productivity are significantly hindered by high temperatures and low precipitation [3–5]. These climatic stressors contribute to decreased yield quantity and quality, as well as increased incidence of fungal diseases [6]. In Thailand, the dry season spans from February to June, during which cacao production faces serious challenges, such as ingrown and undersized pods that weigh less than 350 g, which result in reduced market value and farmer income. This issue is particularly evident in Nan Province, one of the largest cacao-growing regions in Thailand, covering approximately 685 acres [7]. Field observations from this area have reported that up to 60% of cacao pods are either malformed or underweight, making them unsuitable for cacao bean fermentation and commercial processing. Most substandard cacao products are unsellable and are typically discarded, spoiling and producing olfactory pollution in surrounding areas. Despite being unfit for chocolate production, these unqualified cacao pods still contain significant levels of nutritional content and bioactive phytochemicals, including alkaloids, theobromines, saponins, polyphenols, flavonoids, and tannins [8]. Based on the aforementioned properties, the compounds present in substandard cacao may exhibit potential as functional feed additives for beef cattle. Wang et al [9] found that cacao by-products are rich in phenolic compounds known for their antimicrobial and antioxidant properties, which may benefit animal health, digestion, and inflammation control, and reduce gas production. However, the use of fresh cacao poses practical limitations, particularly in terms of storage and transportation. Consequently, we have sought to develop a more stable and user-friendly form that retains its beneficial effects on cattle productivity and health. In this study, the unqualified cacao pods were processed into dried powder and used as an ingredient in mineral feed blocks for cattle. This approach aims to add economic value to cacao by-products while evaluating their effects on cattle productivity.

A major source of methane (CH_4_) and carbon dioxide (CO_2_) production is the livestock industry, a significant contributor to global greenhouse gas emissions, with an estimated global warming potential accounting for 18% to 33% of total CH_4_ emissions [10,11]. Approximately 2% to 12% of total energy intake in ruminants is lost as CH_4_ during enteric fermentation [12]. In a similar context, beef cattle, the most abundant ruminant species in Thailand with, an estimated population of 9.9 million head, represent a promising target for the use of dietary supplements with such gas-reducing functions. Recent studies suggest that plant-derived polyphenols can modulate the rumen microbial community, particularly by inhibiting methanogenic archaea, thereby improving rumen fermentation and reducing CH_4_ emissions [13,14]. Bordiga et al [15] reported that polyphenols, particularly tannins, are mainly responsible for the bioactive properties of cacao. Bio-tanins have the potential to affect ruminant performance and product quality by altering the rumen metabolism and decreasing the CH_4_ emission, according to Frutos et al [16]. However, cacao also contains alkaloids, which may cause a reduction in palatability and toxicity symptoms when fed to livestock in large quantities [17]. Therefore, in this study, unqualified cacao pods were applied as an active feed additive to evaluate their effects. This research aims to evaluate the effects of incorporating unqualified cacao pod powder into cattle diets, with a focus on nutrient digestibility, blood metabolites, rumen fermentation, and greenhouse gas (CO_2_ and CH_4_) emissions. This study also seeks to provide a sustainable solution for cacao farmers by transforming otherwise discarded agricultural waste into a value-added product, ultimately supporting both enhanced farmer income and improved beef quality through a low-carbon livestock production model.

## MATERIALS AND METHODS

### Animals, experimental design, and treatments

The study was conducted from November 2024 to February 2025 at the Beef Cattle Research Unit, part of the Department of Animal Science and Fisheries at Rajamangala University of Technology Lanna (Nan Campus), Thailand. All experimental procedures involving animals were reviewed and approved by the Institutional Animal Care and Use Committee (IACUC) of Rajamangala University of Technology Lanna (RMUTL) under Approval No. RMUTL-IACUC 001/2025. The study was performed in strict accordance with the ethical guidelines for animal care and use established by the National Research Council of Thailand (NRCT).

### Animals, housing, and experimental design

Four two-year-old Brahman×Thai native crossbred steers, with a mean initial body weight (BW) of 207.1±45.1 kg (mean±standard deviation), were used in this study. The animals were housed individually in stalls (2.5×4.5 m^2^). The experiment was conducted using a 4×4 Latin square design (LSD), consisting of four experimental periods, each lasting 21 d.

### Diets and feeding management

All animals were fed twice daily at 08:00 and 17:00. The diet consisted of a concentrate mix and rice straw as the roughage source, which were provided separately. The concentrate mix was offered at a rate of 1.5% of BW, and animals had free access to rice straw. The concentrate mix was formulated to meet the protein requirements for beef cattle (200–250 kg), with a targeted average daily gain (ADG) of 1.0 kg/d, according to NRC [18] guidelines. The ingredients and chemical composition of the experimental diets are presented in Table 1. The chemical composition of the concentrate mix, presented in dry matter (DM), was 16.5% CP, 3.4 % ether extract (EE), 29.1% neutral detergent fiber (NDF), 18.5% acid detergent fiber (ADF), and 4.2% ash. The diet provided 2.78 Mcal/kg DM of metabolizable energy (ME) and a total digestible nutrient (TDN) content of 72.8%. The composition of unqualified cacao pod powder was 8.41% CP, 5.5% EE, 40.2% NDF, 18.4% ADF, and 6.4% ash. The tannin and total polyphenol contents in unqualified cacao pod powder were 22.1 and 56.8 g/kg DM, respectively.

The four experimental treatments were as follows: treatment 1: control group, basal diet with no cacao supplement, treatment 2: basal diet supplemented with 50 g/d of dried cacao powder, treatment 3: basal diet with free-choice access to a mineral lick block containing cacao powder (formulated for a target intake of approximately 50 g of cacao powder/d), and treatment 4: basal diet supplemented with 50 g/d of dried cacao powder and free-choice access to the mineral lick block containing cacao powder.

The mineral feed block containing substandard cacao powder was formulated and manufactured at a certified facility (Korat Salt). The approximate composition per kilogram of the block was as follows: 620 g NaCl, 350 g cacao pod powder, 18.5 g vitamins and additives, 5 g Mg, 2.5 g Ca, 1.6 g Fe, 1.5 g P, 0.30 g Cu, 0.21 g Mn, 0.15 g Co, 0.14 Zn and 0.10 g I.

### Sampling, data collection, and chemical analyses

On the first and final day of each 21 d experimental period, cattle were weighed at 07:30 before morning feeding to record their BW, and on the final day of each period, rumen fluid samples (approx. 200 mL) were collected from each steer via a stomach tube at 4 h post-feeding. The pH of the fresh fluid was measured immediately upon collection using a portable pH meter (HI 8424N; HANNA Instruments). The remaining fluid was then strained through four layers of cheesecloth to remove large feed particles. For preservation, a portion of the filtrate was acidified by adding 10 mL of 1 M H_2_SO_4_ to 90 mL of the fluid. These preserved samples were then stored at −20°C until further analysis.

The concentration of ammonia-nitrogen (NH_3_N) in the preserved rumen fluid was determined using the steam distillation method, according to Bremner and Keeney [19]. For volatile fatty acid (VFA) analysis, preserved samples were thawed and centrifuged at 16,000×g for 15 min at 4°C. The resulting supernatant was collected and passed through a 0.45 μm syringe filter. The final filtrate was stored at −20°C until analysis. VFA concentrations were determined via gas chromatography (GC 8890; Agilent Technologies) equipped with a capillary column (molecular sieve 13×, 30/60 mesh; Alltech Associates), following the method described by Cai [20].

Blood samples of approximately 10 mL were collected from the jugular vein at 4 h post-feeding, concurrent with rumen fluid sampling. The samples were processed for two distinct analyses. For hematological analysis, whole blood was collected in tubes containing EDTA as an anticoagulant and subsequently analyzed for white blood cell (WBC), red blood cell (RBC), hemoglobin, hematocrit, and differential neutrophil and lymphocyte counts. For plasma chemistry, separate samples were collected in heparinized tubes and, immediately centrifuged at 500×g for 10 min to harvest the plasma, which was then stored at −20°C. These plasma samples were later analyzed for concentrations of blood urea nitrogen (BUN) and glucose using an automated analyzer (Cobas Integra 400 Plus; Roche Diagnostics).

To determine apparent nutrient digestibility, fecal samples were collected via rectal grab sampling using a 3×5 design (3 samples per day collected during daylight hours over 5 d period, where samples were collected on days 16–20 at 07:00, 12:00, and 17:00 h), according to the method of Velásquez et al [21]. Samples of the feed offered and feces collected were pooled separately for each steer within each period. The pooled feed and fecal samples were dried in a forced-air oven at 60°C for 72 h and subsequently ground for analysis. Dried samples were analyzed for DM, CP, EE, NDF, and ADF [22]. Acid-insoluble ash (AIA) was analyzed in both feed and fecal samples and used as an internal marker to calculate the apparent digestibility of nutrients [23].

### *In vitro* determination of gas and methane production

CH_4_ and CO_2_ production were determined using an *in vitro* gas production technique based on the method of Menke and Steingass [24]. On the final day of each 21 d experimental period, rumen fluid was collected via a suction tube from each of the experimental steers prior to the morning feeding. The rumen fluid from each steer was used as the inoculum for the substrate corresponding to the treatment that each particular steer was consuming. The collected fluid was immediately strained through four layers of cheesecloth and maintained under warm (39°C), anaerobic conditions.

The substrates for the *in vitro* fermentation were the four experimental treatments, which were dried and ground. The concentrate mix included the ingredients shown in Table 1; its chemical composition, presented in DM, was 16.5% CP, 3.4% EE, 29.1% NDF, 18.5% ADF, and 4.2% ash. The diet provided 2.78 Mcal/kg DM of ME and had a TDN content of 72.8%. The experimental diet consisted of a concentrate mix-to-roughage ratio of 60:40. For each of the four treatments, 0.4 g of the corresponding diet was weighed into a 50 mL glass incubation bottle, with 12 replicate bottles prepared per treatment. All bottles were added with unqualified cacao powder and unqualified cacao powder combined with mineral block supplement, according to the details specified in each experimental treatment (treatment 1; non-supplemented, treatment 2; unqualified cacao powder 0.003 g, treatment 3; mineral block powder 0.01 g, treatment 4; unqualified cacao powder 0.003 g and mineral block powder 0.01 g). A buffer solution (artificial saliva) was prepared by mixing a macro-mineral solution (200 mL), micro-mineral solution (0.1 mL), buffer solution (200 mL), and resazurin indicator solution (1 mL) with distilled water (500 mL). This mixture was maintained under anaerobic conditions by continuously bubbling CO_2_ gas through it until the resazurin indicator turned from pink to colorless. Each incubation bottle containing the substrate was pre-warmed to 39°C before adding 30 mL of an inoculum—buffer mixture (consisting of 10 mL of the appropriate rumen fluid and 20 mL of buffer). The bottles were then immediately sealed and placed in a temperature-controlled incubator at 39°C. Total gas production was recorded at 6, 12, 24, 30, 36, 42, and 48 h. post-incubation. At each time point, the gas pressure in each bottle was measured with a pressure-calibrated syringe to determine the cumulative gas volume.

For CH_4_ production, four bottles per treatment were randomly selected at 24 and 48 h. of incubation time, and a sample of the accumulated gas was collected from each bottle into a vacuum tube for analysis of CH_4_ and CO_2_ concentrations using a gas chromatograph (GC-2014; Shimadzu). The CH_4_ concentration was determined as follows:


(1) $${\text{CH}}_{4}\;(\text{mL})=\text{Total gas produced }(\text{mL})\times \%\;\text{Methane in the sample}$$

*In vitro* organic matter digestibility (IVOMD%) was assessed as IVOMD = 14.88+0.889 GV+0.45 CP+0.651 XA [24], where GV, CP, and XA are total gas volume, crude protein, and ash, respectively. The values of methane production (mL) and grams of IVOMD were used to calculate methane yield, expressed as milliliters per gram of DM and per gram of IVOMD.

### Statistical analysis

The data were analyzed for variance (analysis of variance, ANOVA) using a LSD. The statistical model used was: Y_ijk_ = μ+R_i_+C_j_+T_k_+ɛ_ijk_, where: Y_ijk_ is the observed value, μ is the overall mean, R_i_ is the effect of the animals, C_j_ is the effect of the period, T_k_ is the effect of the treatment, and ɛ_ijk_ is the random error term.

The mean differences between treatments were compared using Tukey’s HSD test, with statistical analysis performed using the software program SAS [25]. The difference between means was considered statistically significant when the p-value was lower than 0.05 (p<0.05), and showed a trend toward a difference in treatment means when the p-value is between 0.05 and 0.10 (0.05<p<0.10).

## RESULTS

### Nutrient intake and apparent digestibility

Supplementation with the cacao pod-based mineral block did not affect concentrate mix, roughage, or total feed intake. Across all treatments, daily intakes ranged from 3.51 to 3.60 kg for concentrate mix, 3.44–3.92 kg for roughage, and 6.95–7.48 kg for total feed (Table 2). Similarly, the daily intakes of DM, organic matter (OM), CP, EE, NDF, and ADF did not differ among the treatments. However, as expected, the daily intake of tannins and polyphenols was higher (p<0.01) in the groups receiving cacao supplements, with intake ranges of approximately 0.66–1.76 g/d and 1.70–4.54 g/d, respectively.

In contrast to the intake results, cacao pod supplementation had a strong positive effect on nutrient digestibility. Compared to the control group, animals in all three cacao-supplemented treatments showed higher apparent digestibility of DM, OM, CP, NDF, and ADF (p<0.01) (Table 3).

### Rumen fermentation efficiency

Supplementation with cacao pod increased ruminal pH; the mean pH was 6.45 for the control group compared to a range of 6.64–6.66 for the cacao-supplemented groups (Table 4). In contrast, the concentration of ruminal NH_3_-N did not differ among treatments. The absolute concentrations of VFA were affected by the dietary treatments. Specifically, cacao supplementation resulted in higher total VFA and acetate (C2) concentrations (p<0.05), while the concentrations of propionate (C3) and butyrate (C4) remained unchanged. However, when expressed as molar proportions, there were no differences, as demonstrated by the acetate-to-propionate ratio (C2:C3), which did not differ among treatments and ranged from 3.20 to 3.43 (Table 4).

### Blood metabolites

The effects of cacao pod supplementation on blood parameters are presented in Table 5. Plasma glucose concentrations were not different among treatments, with values ranging from 61.7 to 66.0 mg/dL. In contrast, BUN levels were lower in animals receiving cacao supplements (15.1–16.9 mg/dL) compared to the control group (18.9 mg/dL) (p<0.05).

For hematological parameters, RBC counts, hemoglobin concentrations, and hematocrit values did not differ among treatments. Similarly, total WBC counts, neutrophil and lymphocyte percentages, and the neutrophil- to- lymphocyte (N:L) ratio were not affected. However, there was a statistical tendency towards a decrease in the supplemented groups for the total WBC count (p = 0.09), the neutrophil percentage (p = 0.06), and the N:L ratio (p = 0.08).

### Methane and carbon dioxide production

The results of the *in vitro* gas production assay, including the total gas volume, CH_4_ and CO_2_ concentrations, and the CH_4_ to CO_2_ ratio, are presented in Table 6. There was a tendency for total gas production to be higher in the cacao-supplemented treatments at both 24 h (p = 0.07) and 48 h (p = 0.06) of incubation. An effect was observed on the concentration of specific gases. Fermentation of diets containing cacao supplements resulted in lower concentrations of both CH_4_ and CO_2_ compared to the control. The control treatment produced the highest concentrations of CH_4_ and CO_2_, respectively, at both 24 h (10.9% and 81.8%) and 48 h (16.3% and 80.8%). Furthermore, the ratio of methane to carbon dioxide (CH_4_/CO_2_) was lower in all cacao-supplemented groups. The mean CH_4_/CO_2_ ratios for these groups ranged from 0.07 to 0.08 at 24 h and 0.13 to 0.17 at 48 h.

CH_4_ production, expressed as mL per gram of DM, was affected by treatment at both 24 and 48 h of incubation. At 24 h, the control group produced a higher amount of CH_4_ (5.57 mL/g DM) than the supplemented group (T2, T3, and T4; 4.35, 4.58, and 3.80 mL/g DM, respectively). At 48 h, the control group exhibited the highest CH_4_ production (12.61 mL/g DM), followed by T3, T2, and T4 (10.1, 8.85, and 7.27 mL/g DM), with all values different from each other. According to CH_4_ production expressed as mL per gram of IVOMD, at 24 h, the yield of CH_4_ in the control group (0.97) was higher than T2 (0.80), T3 (0.81), and T4 (0.72). At 48 h, CH_4_ production was highest in the control group (2.65 mL/g IVOMD), followed by T3 (2.17), T2 (1.96), and T4 (2.17, 1.96 and 1.66 mL/g IVOMD), with treatments resulting in differences.

## DISCUSSION

Based on the results of this study, the supplementation of unqualified cacao pods in all treatments had no effect on feed intake in beef cattle but resulted in improved nutrient digestibility. The supplementation of unqualified cacao pods and unqualified cacao pods in mineral blocks did not negatively affect feed intake in beef cattle. Recent studies show that moderate levels of inclusion of polyphenol-rich by-products do not impair palatability or voluntary intake in ruminants [26,27]. No significant difference in intake levels in this experiment suggests that cacao pod-derived tannins and polyphenols, at the concentrations provided, did not have adverse effects that typically reduce feed consumption at higher dosages. Unqualified cacao pod supplementation significantly improved the apparent digestibility of DM, OM, CP, NDF, and ADF. This improvement can be attributed to the bioactive compounds in cacao pods, particularly condensed tannins and polyphenols. The modulation of ruminal microbial populations by polyphenols may favor fibrolytic bacteria, leading to enhanced fiber degradation and VFA production [28,29]. In addition, Carrasco et al [30] reported that supplementation with a mixture of chestnut tannin at 0.2% in the diet enhanced fibrolytic, amylolytic, and ureolytic bacterial communities in the rumen and reduced methanogenic archaea. In a study by Prapaiwong et al [31], the cows given hydrolyzable tannin showed an increase in the digestibility of DM, OM, and CP. Additionally, supplementation of cacao pod powder or cacao-enriched mineral blocks may appear to stimulate salivary secretion, leading to the maintenance of ruminal pH conducive to microbial activity. According to the present study, beef cattle supplemented with unqualified cacao pods exhibited higher ruminal pH levels compared to the control group. Although several reports have indicated that supplementation of polyphenols in a ruminant diet may tend to reduce ruminal fermentation, their use at low levels has been found to have a positive effect on ruminal digestibility. Moreover, overall digestibility throughout the gastrointestinal tract tends to improve. This is consistent with the findings of Aguiar et al [32], who reported that supplementation with 5.1–6.2 g of phenolic compounds/d reduced ruminal digestibility, while supplementation with 2.95 g of phenolic compounds/d had no adverse effect on ruminal digestibility. However, all levels of supplementation resulted in increased total digestibility of DM, OM, NDF, and overall digestibility. These findings are consistent with the results of the present study, which showed that supplementation with unqualified cacao pods in all treatments provided a total polyphenol intake of 1.70–4.54 g/head/d. This level did not affect ruminal digestibility but positively influenced the overall nutrient digestibility throughout the gastrointestinal tract.

The supplementation of unqualified cacao pods increased ruminal pH. This finding is consistent with previous studies reporting that polyphenol supplementation stabilized ruminal pH and reduced the accumulation of lactic acid [33]. Importantly, the total VFA and acetate concentrations were enhanced without altering the acetate-to-propionate ratio, suggesting that fiber fermentation was improved without shifting the overall fermentation pathway toward less energy-efficient routes [26]. The elevated ruminal pH observed in cacao-supplemented groups is beneficial for fiber degradation. Optimal fiber digestion by cellulolytic bacteria requires a ruminal pH above 6.2 [34,35]. Reiche et al [28] reported that dietary dried cacao bean shell (5% of the diet formula) in dairy cows showed higher ruminal pH than the control group (6.32 vs. 6.16). Therefore, a higher ruminal pH in cacao pod-supplemented cattle likely created a more favorable environment for fibrolytic microbial growth and enzymatic activities, explaining the observed enhancements in the digestibility of DM, OM, NDF, and ADF. This suggests that cacao supplementation not only influences fermentation end-products but also strategically improves fiber utilization and overall ruminal efficiency.

In the blood metabolite profiles, glucose, WBC counts, RBC counts, hemoglobin concentration, and hematocrit values remained within normal physiological ranges across all treatments. These results confirm the safety of cacao pod supplementation at the tested levels, with no indications of systemic inflammation or hematological toxicity. However, this study found that the total WBC count, neutrophil percentage, and N:L ratio tended to decrease in the supplemented groups. A reduction in the total WBC count and neutrophil was observed in cattle supplemented with cacao, which is rich in tannins and polyphenols. This may reflect a reduction in systemic inflammation or immune activation. Neutrophils are the first line of defense in the innate immune system and are typically elevated in response to infection, inflammation, or stress [36]. Therefore, a lower neutrophil count in this context indicates that the animals had less inflammation, possibly due to the antimicrobial effects of polyphenols in the gut or improved rumen health. Moreover, polyphenols are known to inhibit the production of proinflammatory cytokines [37], which can lead to decreased regulation of immune cell activation and recruitment, thereby reducing the number of WBCs.

Interestingly, BUN levels were significantly decreased in the supplemented animals. The reduction in BUN may reflect more efficient utilization of rumen ammonia due to improved microbial protein synthesis and reduced proteolysis by rumen microbes, consistent with the observed improvements in CP digestibility [38]. Although the ruminal NH_3_-N concentrations did not differ among treatments, the observed values tended to decrease in all treatments supplemented with unqualified cacao pods. The reduction in BUN indicates improved utilization of ruminal protein, resulting in decreased ammonia absorption into the bloodstream. Tannins or polyphenols present in cacao bind to protein molecules, resulting in reduced protein degradation in the rumen. Consequently, more protein bypasses the rumen and passes into the abomasum and small intestine, where it is directly digested by the animal’s endogenous enzymes. Carrasco et al [30] and Prapaiwong et al [31] support these findings. Dietary inclusion of chestnut or quebracho tannins (3–9 g/d) in cattle diets showed lower ruminal and plasma ammonia levels, accompanied by improved nitrogen retention and reduced urinary nitrogen excretion.

A remarkable outcome of the present study is the substantial reduction in CH_4_ and CO_2_ production in animals supplemented with, on average, 50 g/head/d of cacao pod powder, receiving approximately 0.66–1.76 and 1.70–4.54 g/d of tannins and polyphenols, respectively. Notably, recent meta-analyses confirm that dietary tannins at appropriate levels can consistently lower enteric CH_4_ production without compromising animal performance [39]. The reduction in methane is likely linked to the direct inhibition of methanogenic archaea and the decrease in protozoal populations mediated by tannins, as previously reported [27]. Condensed tannins can bind to microbial cell walls, disrupt membrane integrity, and reduce hydrogen availability for methanogenesis [29].

## CONCLUSION

Supplementation with unqualified cacao pod or unqualified cacao pod in mineral blocks approximately receiving 50 g/head/d of cocoa powder in beef cattle did not negatively affect feed intake but significantly improved nutrient digestibility and rumen fermentation efficiency in beef cattle. This was further supported by a decrease in BUN levels, indicating more effective nitrogen utilization. Crucially, *in vitro* analysis demonstrated that this supplementation also led to a reduction in CH_4_ and CO_2_ production. Collectively, these findings indicate that substandard cacao pod is a promising and sustainable feed additive that can enhance digestive function in beef cattle with the added potential of mitigating greenhouse gas emissions.

## Figures and Tables

**Table 1:** Feed ingredients and chemical composition of concentrate mix, rice straw and UCP

Ingredients (% DM)	Concentrate mix	Rice straw	UCP
Cassava chip	42.0		
Corn meal	10.0		
Rice bran	8.50		
Rice bran with hull	10.0		
Palm kernel cake	18.0		
Soybean meal	8.00		
Urea	1.80		
Sulfur	0.20		
Salt	0.3		
Dicalcium phosphate	1.00		
Premixed	0.20		
Chemical composition			
Dry matter (%)	90.8	91.4	92.3
Organic matter (% of DM)	86.6	78.9	85.9
Crude protein (% of DM)	16.5	2.84	8.41
Ether extracts (% of DM)	3.40	1.12	5.50
Neutral detergent fiber (% of DM)	29.1	78.9	40.2
Acid detergent fiber (% of DM)	18.5	54.8	18.4
Ash (% of DM)	4.20	12.5	6.40
Metabolizable energy (Mcal/kg of DM)	2.78	1.46	-
Total digestible nutrient (TDN, %)	72.8	45.4	-
Tannin (g/kg DM)	-	-	22.1
Polyphenol (g/kg DM)	-	-	56.8

UCP, undergrade cacao pod; DM, dry matter.

**Table 2:** Effect of unqualified cacao pod in mineral feed block on intake in beef cattle

Items	Treatment				SEM	p-value

	T1	T2	T3	T4		
Average body weight (BW, kg)	228.2	238.5	233.1	238.4	-	-
Feed intake						
Concentrates (kg/d)	3.53	3.51	3.56	3.50	0.25	0.40
Concentrates (% BW)	1.54	1.48	1.52	1.48	0.39	0.35
Roughage (kg/d)	3.60	3.65	3.72	3.64	0.20	0.11
Total intake (kg/d)	7.13	7.16	7.28	7.14	1.34	0.38
Total intake (% BW)	3.13	3.00	3.13	2.99	0.53	0.17
Total intake (g/kg BW^0.75^)	121.5	117.9	122.1	117.7	24.2	0.18
Nutrients intake (kg DM/d)						
DM	6.50	6.33	6.82	6.50	1.22	0.16
Organic matter	5.90	5.75	6.18	5.90	1.54	0.14
Crude protein	0.69	0.68	0.70	0.68	0.33	0.12
Ether extract	0.16	0.16	0.16	0.16	0.55	0.98
Neutral detergent fiber	3.83	3.74	4.13	3.89	2.21	0.22
Acid detergent fiber	2.60	2.53	2.81	2.64	1.01	0.39
Unqualified cacao pod (g/d)	0.00^a^	50.0^b^	31.0^b^	81.6^b^	12.4	0.01
Tannin (g/d)	0.00^a^	1.11^b^	0.66^b^	1.76^b^	0.35	0.01
Polyphenol (g/d)	0.00^a^	2.84^b^	1.70^b^	4.54^b^	0.44	0.01

a,b Means with different superscripts in the same row are different (p<0.05).

T1, mineral block with no supplement cacao powder; T2, mineral block with dried cacao powder 50 g/d; T3, mineral mixed cacao lick block (approximate 50 g/d); T4, dried cacao powder 50 g/d+mineral mixed with cacao lick block (approximate 50 g/d); SEM, standard error of the mean; DM, dry matter.

**Table 3:** Effect of unqualified cacao pod in mineral feed block on digestibility in beef cattle

Items	Treatment				SEM	p-value

	T1	T2	T3	T4		
Dry matter (%)	64.7^c^	67.1^b^	71.0^a^	71.8^a^	1.40	<0.01
Organic matter (%)	68.6^c^	71.3^b^	75.4^a^	74.8^a^	1.05	<0.01
Crude protein (%)	64.2^c^	68.6^ab^	70.3^ab^	73.7^a^	1.03	<0.01
Ether extract (%)	88.4	87.7	87.4	90.0	1.37	0.10
Neutral detergent fiber (%)	56.9^b^	61.8^ab^	65.8^a^	68.5^a^	1.18	<0.01
Acid detergent fiber (%)	36.6^c^	40.3^bc^	45.3^b^	51.8^a^	1.14	<0.01

a–c Means with different superscripts in the same row are different (p<0.05)

T1, mineral block with no supplement cacao powder; T2, mineral block with dried cacao powder 50 g/d; T3, mineral mixed cacao lick block (approximate 50 g/d); T4, dried cacao powder 50 g/d+mineral mixed with cacao lick block (approximate 50 g/d); SEM, standard error of the mean.

**Table 4:** Effect of unqualified cacao pod in mineral feed block in beef cattle fed on rumen fermentation efficiency

Items	Treatment				SEM	p-value

	T1	T2	T3	T4		
Rumen fermentation, 4 h after feeding						
pH	6.45^a^	6.65^b^	6.66^b^	6.64^b^	0.12	0.04
NH_3_N (mg/dL)	15.8	14.5	14.2	14.2	0.81	0.35
VFAs (mmol/L)						
Total VFAs	106.2^a^	119.7^b^	116.0^b^	125.6^b^	7.01	0.01
Acetate (C2)	71.9^a^	82.0^b^	81.2^b^	88.2^b^	3.44	0.05
Propionate (C3)	22.0	25.6	24.0	25.7	4.32	0.19
Butyrate (C4)	12.3	12.1	10.8	11.7	1.01	0.11
VFAs (mol/100 mol)						
Acetate (C2)	67.7	68.5	70.0	70.2	5.88	0.69
Propionate (C3)	20.7	21.4	20.7	20.5	2.54	0.31
Butyrate (C4)	11.6	10.1	9.32	9.28	1.26	0.72
C2:C3	3.26	3.20	3.38	3.43	1.01	0.15

a,b Means with different superscripts in the same row are different (p<0.05).

T1, mineral block with no supplement cacao powder; T2, mineral block with dried cacao powder 50 g/d; T3, mineral mixed cacao lick block (approximate 50 g/d); T4, dried cacao powder 50 g/d+mineral mixed with cacao lick block (approximate 50 g/d); SEM, standard error of the mean; NH_3_N, ammonia nitrogen; VFAs, volatile fatty acids.

**Table 5:** Effect of unqualified cacao pod in mineral feed block on blood metabolite in beef cattle

Hematology profile	Treatment				SEM	p-value

	T1	T2	T3	T4		
Glucose (mg/dL)	61.6	66.0	61.7	63.8	3.01	0.25
Blood urea nitrogen (mg/dL)	18.9^b^	16.9^a^	15.0^a^	16.9^a^	1.34	0.03
White blood cell (cell/mm^3^)	10,300	10,400	9,233	9,850	102.4	0.09
Red blood cell (10^6^ cell/mm^3^)	6.16	6.21	6.05	5.97	2.11	0.18
Hemoglobin (g/dL)	8.68	9.53	9.13	8.23	2.61	0.21
Hematocrit (%)	27.3	31.0	30.1	27.5	4.25	0.42
Neutrophill (%)	34.8	32.7	33.0	31.5	0.98	0.06
Lymphocyte (%)	47.8	49.7	47.8	46.0	1.91	0.22
N:L Ratio	0.73	0.66	0.69	0.68	0.03	0.08

a,b Means with different superscripts in the same row are different (p<0.05).

T1, mineral block with no supplement cacao powder; T2, mineral block with dried cacao powder 50 g/d; T3, mineral mixed cacao lick block (approximate 50 g/d); T4, dried cacao powder 50 g/d+mineral mixed with cacao lick block (approximate 50 g/d); SEM, standard error of the mean; N:L Ratio, neutrophill to lymphocyte ratio.

**Table 6:** Effect of unqualified cacao pod in mineral feed block on methane and carbon dioxide production

Hematology profile	Treatment				SEM	p-value

	T1	T2	T3	T4		
Gas production (mL/0.4 g DM)						
24 h	20.7	23.5	21.4	24.9	2.13	0.07
48 h	31.0	34.2	32.5	36.1	2.28	0.06
CH_4_ production (%)						
24 h	10.8^b^	7.39^a^	8.55^ab^	6.11^a^	1.04	0.01
48 h	16.3^b^	10.4^a^	12.4^a^	8.06^a^	1.14	0.01
CO_2_ production (%)						
24 h	81.8^b^	69.8^a^	71.9^a^	64.3^a^	3.12	0.01
48 h	80.8^b^	71.8^a^	75.9^a^	71.9^a^	4.62	0.01
CH_4_/CO_2_ ratio						
24 h	0.13	0.11	0.11	0.10	0.03	0.12
48 h	0.19^b^	0.14^a^	0.16^a^	0.11^a^	0.03	0.01
CH_4_ production (mL/gDM)						
24 h	5.57^b^	4.35^a^	4.58^a^	3.80^a^	0.28	0.01
48 h	12.6^c^	8.85^a^	10.1^b^	7.27^a^	0.369	0.01
CH_4_ production (mL/IVOMD)						
24 h	0.97^b^	0.80^a^	0.81^a^	0.72^a^	0.03	0.01
48 h	2.65^c^	1.96^a^	2.17^b^	1.66^a^	0.07	0.01

a–c Means with different superscripts in the same row are different (p<0.05).

T1, mineral block with no supplement cacao powder; T2, mineral block with dried cacao powder 50 g/d; T3, mineral mixed cacao lick block (approximate 50 g/d); T4, dried cacao powder 50 g/d+mineral mixed with cacao lick block (approximate 50 g/d); SEM, standard error of the mean; DM, dry matter; IVOMD, *in vitro* organic matter digestibility.

## Data Availability

Upon reasonable request, the datasets of this study can be available from the corresponding author.
